# A high visibility Talbot-Lau neutron grating interferometer to investigate stress-induced magnetic degradation in electrical steel

**DOI:** 10.1038/s41598-020-58504-7

**Published:** 2020-02-04

**Authors:** Tobias Neuwirth, Alexander Backs, Alex Gustschin, Simon Vogt, Franz Pfeiffer, Peter Böni, Michael Schulz

**Affiliations:** 1 Technical University of Munich, Heinz Maier-Leibnitz Zentrum (MLZ), Lichtenbergstr. 1, 85748 Garching, Germany; 2 Technical University of Munich, Department of Physics, Chair for Neutron Scattering (E21), James-Franck-Str. 1, 85748 Garching, Germany; 3 Technical University of Munich, Department of Physics and Munich School of Bioengineering, Chair of Biomedical Physics, James-Franck-Str. 1, 85748 Garching, Germany; 4 Technical University of Munich, Chair of Metal Forming and Casting, Walther-Meißner-Str. 4, 85748 Garching, Germany; 5 Technical University of Munich, Department of Diagnostics and Interventional Radiology, Klinikum rechts der Isar, Ismaninger Str. 22, 81675 Munich, Germany

**Keywords:** Ferromagnetism, Magnetic properties and materials, Imaging techniques

## Abstract

Neutron grating interferometry (nGI) is a unique technique allowing to probe magnetic and nuclear properties of materials not accessible in standard neutron imaging. The signal-to-noise ratio of an nGI setup is strongly dependent on the achievable visibility. Hence, for analysis of weak signals or short measurement times a high visibility is desired. We developed a new Talbot-Lau interferometer using the third Talbot order with an unprecedented visibility (0.74) over a large field of view. Using the third Talbot order and the resulting decreased asymmetry allows to access a wide correlation length range. Moreover, we have used a novel technique for the production of the absorption gratings which provides nearly binary gratings even for thermal neutrons. The performance of the new interferometer is demonstrated by visualizing the local magnetic domain wall density in electrical steel sheets when influenced by residual stress induced by embossing. We demonstrate that it is possible to affect the density of the magnetic domain walls by embossing and therefore to engineer the guiding of magnetic fields in electrical steel sheets. The excellent performance of our new setup will also facilitate future studies of dynamic effects in electric steels and other systems.

## Introduction

Neutron radiography is a method allowing for non-destructive analysis of the inner structure of an object^1^. Because the neutron cross-sections show no systematic dependence on the atomic number, both light and heavy elements can be visualized. Moreover, the contrast between different materials can be varied by using isotopes. Therefore, neutron imaging has been established to be a very efficient technique in materials science, research in cultural heritage, archaeology, and engineering, where imaging with X-rays fails to produce sufficient contrast. Neutron imaging is, however, limited by the coarse spatial resolution imposed by the limitations in neutron flux and the spatial resolution of the neutron detectors. Currently, the achieved spatial resolution is in the low single μm range^2–7^. Paths towards resolving structures with higher resolution (e.g. water transport in fuel cells) are, for example, improving the detector resolution^3–6^ or in some cases using neutron grating interferometry (nGI)^8,9^ as a spatially resolved ultra-small-angle scattering technique. nGI simultaneously gathers spatially resolved information about the transmission- (TI), the differential phase contrast- (DPCI) and the scattering/dark-field (DFI) of a sample. Most notably, the contrast provided by the DFI^10,11^ is generated by ultra-small-angle neutron scattering (USANS) off structures on a length scale similar to the correlation length of the interferometer setup, which is typically in the range 0.1 μm to 10 μm. Such structures are caused by variations of the nuclear or magnetic scattering length density. nGI has been used for the analysis of material structures and the investigation of magnetic domains in ferromagnets^12^ and vortex lattice domains in superconductors^13^.

In recent years neutron grating interferometers deviating from the highly asymmetric Talbot-Lau geometry introduced by Pfeiffer *et al*.^14^ have been built. For Talbot-Lau types of interferometers higher Talbot orders have been used. This allows easier positioning of samples between G_1_ and G_2_, as well as tuning of the correlation length without changing the neutron wavelength^8,15^. Typically a visibility of 0.25 has been achieved. Also symmetric Talbot-Lau interferometers have been introduced^16,17^, which have typically a higher maximum phase sensitivity than an asymmetric setup. A visibility of 0.2 has been reported^17^. In contrast to the above considered variations of Talbot-Lau interferometers, the concept of purely phase-grating based neutron far-field interferometry was introduced. Interferometers with two^18^ or three^19^ phase-gratings were shown. Visibilities of 0.13 and 0.32 have been reached, respectively.

At the same time, nGI has started to transform from a qualitative tool sensitive to the presence of microstructures to a quantitative tool, also providing detailed information on the size and the orientation of microstructures inside a sample^20–24^. An advantage for a quantitative analysis of these parameters is a high contrast of the interference pattern generated by the nGI-setup to decrease the error accrued during the measurement. Hence, the contrast produced by the nGI, denoted as visibility, may be used as a figure of merit^25^.

**Figure 1:**
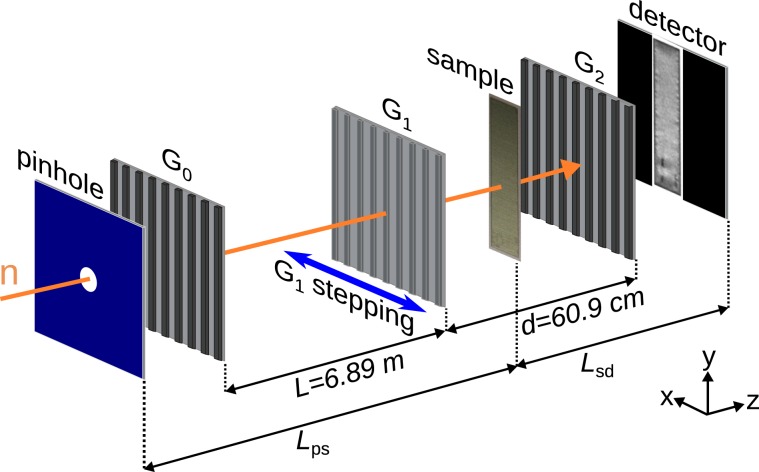
Schematics of the neutron grating interferometer (nGI) -setup. It is based on two absorbing gratings G_0_ (source) and G_2_ (analyzer), and a phase grating G_1_. The sample is typically placed in the space between G_1_ and G_2_.

Here we report on the design and the application of a newly designed neutron grating interferometer using Talbot-Lau interferometry, which has recently been implemented at the ANTARES imaging beamline at the Heinz Maier-Leibnitz Zentrum (MLZ)^26,27^. A schematic view of the interferometer is shown in Fig. 1. The setup has been tuned towards a more symmetric geometry compared to our previous design^22^ and other similar existing nGI setups in asymmetric Talbot-Lau geometry^28,29^. The third Talbot order has been used to achieve this decreased asymmetry. The total length of the setup is approximately 8 m and the distance *d* between G_1_ and G_2_ has been significantly increased from ≈2 cm to ≈61 cm. This change of geometry allows to use a larger G_2_ period, enabling the fabrication of a highly absorbing binary G_2_-grating by using a newly developed technique of Gd powder deposition into an etched Si grating structure^30^. The improved quality of the G_2_-grating is the hallmark towards achieving a high visibility^25^.

The new interferometer reaches a visibility of $V\approx 0.74$ at 4 Å over the whole field of view (FoV) of the detector system (71 mm × 76 mm) which is the highest *V* achieved on such a large FoV. At 5 Å a comparable visibility *V* ≃ 0.69 has been reported by Seki *et al*.^31^, however, due to the metallic glass imprinting process used for the fabrication of the G_2_-grating only small FoVs (10 mm × 10 mm) have been realized until now. Most gratings are currently produced by sputtering or evaporation of Gd under an inclined angle onto an etched Si line grating^22,29,32^ and a visibility *V* of ≈0.25 over a FoV of 64 mm × 64 mm is typically achieved. As a result of the highly improved visibility over a large FoV, experiments with samples exhibiting only small variations in scattering contrast or requiring short measurement times now become more accessible. In this work we demonstrate the capabilities of our novel nGI setup to study the magnetic domain structure of non grain-oriented electrical steel sheets^33^. Typically, highly performing electric motors are limited in rotational speed and efficiency by the cut-outs machined into their magnetic core, which are required to guide the magnetic flux^34,35^. We propose the novel approach of embossing the sheets to induce stress allowing to pin the magnetic domains using inverse magnetostriction^36,37^. Due to the pinning of the magnetic domains, the magnetic permeability of the material is decreased in the area of the applied stress. This allows to guide the magnetic flux without the introduction of cut-outs in the magnetic core. As a result of the improved mechanical stability higher rotational speeds may be used, thus increasing the efficiency. To analyze the effect of stress in the bulk of the material, the bulk magnetic domain structure has to be mapped. This is not possible with standard (surface sensitive) techniques such as magneto-optical Kerr effect (MOKE)^38^, as the surface magnetic domains may vary strongly with respect to the bulk magnetic domains. Using the new nGI-setup we investigate the pinning of the domain walls in the bulk material to quantify the effect of stress that is induced by embossing.

## Results

### Simulations of the visibility

**Figure 2:**
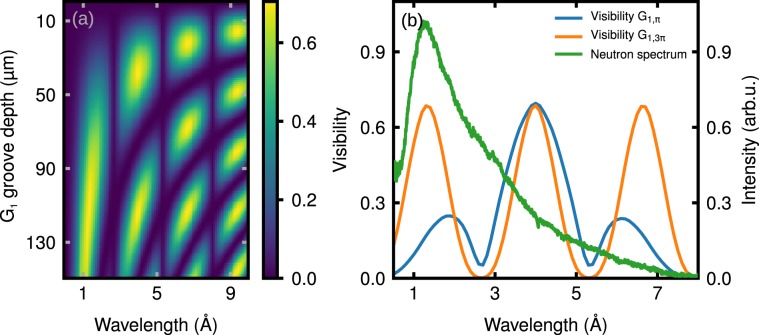
(**a**) Simulation of the visibility of the new setup depending on the groove depth *h*_1_ of the phase grating G_1_ and the wavelength λ. (**b**) Plotted spectral visibilities of *π*-phase-shifting (blue line) and 3*π*-phase-shifting (orange line) setups and the ANTARES neutron spectrum (green line). A 3 *π*-grating allows using the setup also in the sub-2 Å regime, where the spectrum at ANTARES is most intense.

To develop and optimize the new design of the nGI setup we implemented a wave-optical simulation software based on a Fresnel-propagator^39^ and numerical convolution calculations, which quantitatively describe the formation of the fringe contrast that is created by the three gratings G_0_, G_1_, and G_2_^25,40^. We determined the visibility depending on the groove depth *h*_1_ of the silicon grating G_1_ and the wavelength *λ*. For the simulations, the duty cycles (fraction of transparent area) of the gratings G_0_ and G_2_ were set to 0.3 and 0.5, respectively. The results shown in Fig. 2(a) indicate spots of high visibility related to odd multiples of *π*-phase shifts. Around *λ* = 4 Å and *h*_1_ = 38 μm and 114 μm, pronounced maxima in *V* appear. The respective gratings, which shift the neutron waves by *π* and by 3*π*, respectively, are denoted in the following by G_1,__π_, and G_1,3__π_.

Because the phase shift of the neutron waves is proportional to *λ*, the gratings also offer a high visibility for other wavelengths. According to Fig. 2(b), grating G_1,3π_ could be also used as *π* and 5*π* phase-shifting for wavelengths *λ* = 1.3 Å and *λ* = 6.7 Å, respectively. As expected from theory^41,42^, at even integers of fractional Talbot distances there is no intensity modulation over the entire groove depth range of G_1_.

Figure 2(b) shows the visibility vs. *λ* of the gratings G_1,π_ (blue line) and the G_1,3π_ (orange line) together with the spectrum of ANTARES in arbitrary units (green line). The graph illustrates the benefit of using G_1,3π_ at neutron wavelengths in the sub-2 Å regime yielding a high visibility, as G_1,3π_ acts in this regime as a *π* phase-shifting grating. G_1,3π_ performs also excellently above 6 Å, i.e. much better than G_1,π_. In contrast, G_1,π_ exhibits a broader visibility peak at 4 Å and is therefore better suited if a broad wavelength band is available. Considering the results of our simulations we decided to implement the option of exchanging the two phase gratings G_1,π_ and G_1,3π_.

Due to the challenges involved in the fabrication of the gratings, their effective parameters deviate slightly from the design parameters. In particular the duty cycle has a significant influence on the performance of the gratings^25,40^. It is a challenging parameter to control. Therefore, the simulations described in the following, which are compared to experimental data, have been adapted to the effective design parameters, which are listed in Table 1.

**Table 1:** Effective design parameters of the new nGI-setup as realized at ANTARES.

	Parameter	Value
	Design wavelength *λ*	4.0 Å
	*n* (Fractional Talbot order)	3
	*d*	60.9 cm
	*L*	6.89 m
G_0_	*p*_0_	150 μm
	*DC*_0_	0.18 | 0.28 | 0.38 | 0.48
	*GW*_0_	123 μm | 108 μm | 93 μm | 78 μm
	*h*_0_	≈180 μm
G_1_	*p*_1_	24.4 μm
	*DC*_1_	*π*) 0.49 | 3*π*) 0.55
	*GW*_1_	*π*) 12.44 μm | 3*π*) 10.98 μm
	*h*_1_	*π*) 41 μm | 3*π*) 123 μm
G_2_	*p*_2_	13.3 μm
	*DC*_2_	0.45 μm
	*GW*_2_	7.32 μm
	*h*_2_	≈85 μm

*d* and *L* designate the distance between gratings G_1_ and G_2_ and between G_0_ and G_1_, respectively. *p*_*i*_, *DC*_*i*_, *GW*_*i*_, and *h*_*i*_ (*i* = 0,1,2) indicate the period, duty cycle, groove width, and the groove depth of the gratings, respectively.

### Visibility of the setup

To benchmark the performance of the new nGI, we measured the wavelength dependence of the visibility of the nGI-setup using absorption gratings G_0_ with various duty cycles *DC*_0_ and phase shifting gratings inducing phase shifts of *π* and 3*π* (G_1,π_ G_1,3π_), respectively. For a detailed technical description of the new interferometer setup see Sec. 4.3. Details of the experiment, such as the acquisition times, the wavelength ranges, and the resulting peak visibilities of the scans are listed in Table 2.

**Table 2:** Comparison of the simulated with the measured peak visibility of the nGI-setup.

*DC*_0_	G_1_	Wavelength range	h_1_(μm)	Total exposure (s)	λ(Å)	Measurement	Simulation
						Peak visibility	Peak visibility
0.4	*π*	3.0 Å–5.75 Å	43	1280	3.9	0.21	–
0.18	*π*	3.5 Å–4.5 Å	41	240	4.0	0.74	0.80
0.28	*π*	1.6 Å–6.0 Å	41	480	4.0	0.69	0.74
0.38	*π*	3.5 Å–4.5 Å	41	240	4.0	0.63	0.66
0.48	*π*	3.5 Å–4.5 Å	41	240	4.0	0.54	0.57
0.28	3*π*	1.6 Å–6.0 Å	123	480	3.75	0.60	0.61

For the simulations (see Sec. 2.1) the effective parameters listed in Table 1 have been used. The measured visibility of the new setup is significantly larger than the visibility of the previous setup (first row) and approaches the theoretical optimum.

**Figure 3:**
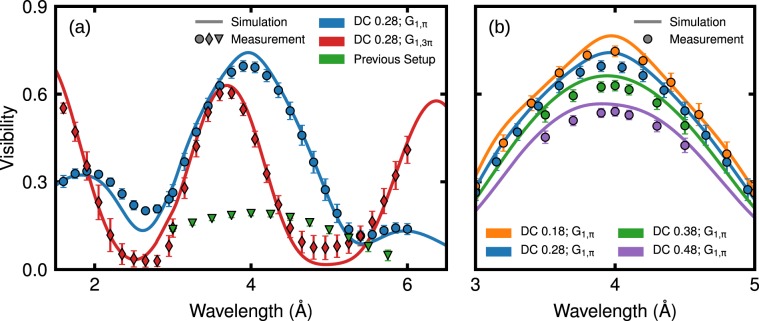
(**a**) Simulated (solid lines) and measured (circle, diamond and triangular markers) wavelength scans of the *π*/3*π* phase grating configurations. The simulations were performed using the actual grating parameters from Table 2 and considering the polychromaticity of the beam. The performance of the previous setup is shown for comparison (triangular markers in green). (**b**) Measured visibilities for different G_0_ configurations for *λ* ≈ 4 Å using G_1,π_ compared with their respective simulations (solid lines).

**Figure 4:**
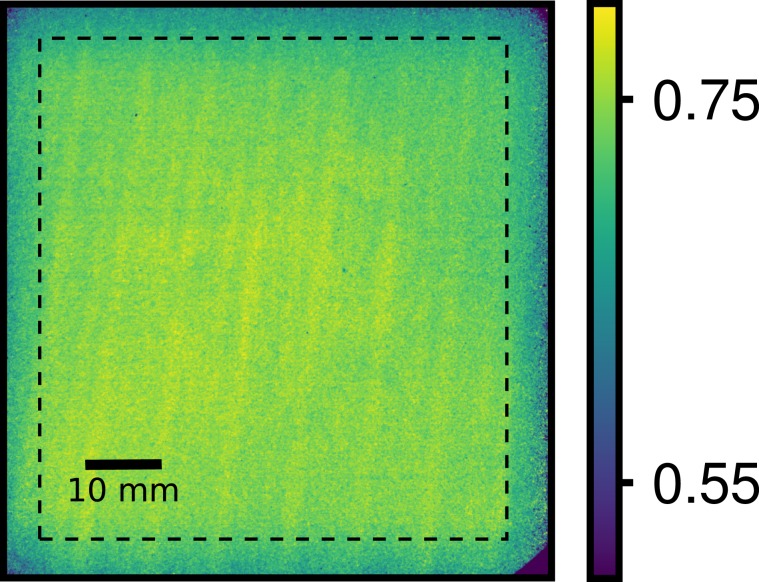
Visibility map of the grating interferometer as determined at a wavelength *λ* = 4 Å using grating G_0_ with a duty cycle *DC*_0_ = 0.18. The average visibility over 75% of the field of view is *V* ≃ 0.74. The area is designated by the dashed line. The visibility degrades slightly towards the boundary of the FoV because the beam size has been limited by an aperture to reduce background.

Figure 3(a) shows the visibility *V* of the new nGI-setup for 1.6 Å ≤ *λ* ≤ 6.0 Å and a fixed duty cycle *DC*_0_ = 0.28 using the gratings G_1,π_ (blue markers) and G_1,3π_ (red markers). *V* was obtained by averaging the data over the central area of 75% of the FoV (Fig. 4). The comparison of *V* with the performance of the previous setup (green markers) demonstrates a tremendous increase in *V*. The new setup exhibits also a reasonably large visibility at the secondary peaks. The higher visibility *V* = 0.35 (grating G_1,π_) at *λ* = 1.8 Å when compared with *V* = 0.14 at *λ* = 5.8 Å is a result of the deviation of the effective groove depth *h*_1_ = 41 μm of G_1,π_ from the nominal design value ${h}_{1}^{nom}\mathrm{=38}\,{\rm{\mu }}{\rm{m}}$. The visibility of the phase grating G_1,3π_ (red markers) shows a peak at *λ* = 3.75 Å with an amplitude of 0.60. The deviation of the peak position from the simulated value *λ* = 4.0 Å (see Fig. 2(b)) is a result of the deviation of the effective groove depth *h*_1_ = 123 μm from the design value ${h}_{1}^{nom}\mathrm{=114}\,{\rm{\mu }}{\rm{m}}$.

The agreement of the simulations and the data using the parameters of the nGI-setup from Table 2 shown in Fig. 3(a) by solid lines is very good. Note that the trapezoidal profile of the etched grooves results in a depth dependent width of the grooves. This effect has not been considered in the simulations. The peak wavelength and peak visibility of the secondary peaks cannot be fully assessed, as the maxima lie outside of the accessible wavelength range. In agreement with Fig. 2(b), a visibility of *V* ≈ 0.56 is reached close to *λ* = 1.6 Å.

Figure 3(b) shows the visibility at *λ* ≈ 4 Å of the new setup using G_1,π_ and gratings G_0_ with four different duty cycles. As expected, *V* increases with decreasing *DC*_0_. A maximum visibility *V* ≈ 0.74 is obtained at *λ* = 4 Å using *DC*_0_ = 0.18. The corresponding visibility map is shown in Fig. 4. *V* is very homogeneous over the whole FoV having a standard deviation of 0.021 inside the marked area. Towards the edges of the FoV a slight decrease of *V* is observed. It is caused by inhomogeneities of the analyzer grating^30^ and the limitation of the beam size with a diaphragm to reduce the background. The measured and simulated peak visibilities for each measurement are given in Table 2 and agree well with each other. For comparison of the new with the previous setup, the *DC*_0_ = 0.38 of the new setup has been used as this is the most similar to the *DC*_0_ = 0.4 of the previous setup, resulting in a similar spatial coherence. In this case, the new setup reaches a visibility of 0.63 at a wavelength of 4.0 Å, while the maximum of the visibility of the old setup is 0.21 at a wavelength of 3.9 Å underlining the outstanding performance of the new gratings.

### Variation of the correlation length

The placement of the sample in the space (60.9 cm) between G_1_ and G_2_ allows for a wide variation of the correlation length $\xi =\lambda {L}_{sd}/{p}_{2}$ (see Sec. 4.2) of the setup, which enables a quantitative analysis of the microstructure within the sample^20,22,24^. Firstly, changing the position of the sample allows operation of the nGI at the design wavelength with constantly high visibility and therefore constantly good signal-to-noise ratio. A trade off of this mode of operation is the variation of the spatial resolution with the sample to detector distance. Assuming a minimum and maximum distance between sample and G_2_ of 2 cm and 50 mm, respectively, the new setup provides a range of correlation lengths of 0.602 μm ≤ *ξ* ≤ 15.038 μm, when operated at the design wavelength *λ* = 4 Å. Secondly, by adjusting *λ* between 1.6 Å and 6 Å and the sample position, the range increases to 0.241 μm ≤ *ξ* ≤ 26.165 μm. For comparison, the previous nGI setup was restricted to a range of 0.796 μm ≤ *ξ* ≤ 2.983 μm. Here the Talbot distance *d* was 19.9 mm, the G_2_ period was 4 μm and the wavelength range was 1.6 Å and 6 Å. The sample to G_1_ distance was kept at a fixed value of 2 cm. The sample was placed between G_0_ and G_1_.

### Stress in electrical steel sheets

**Figure 5:**
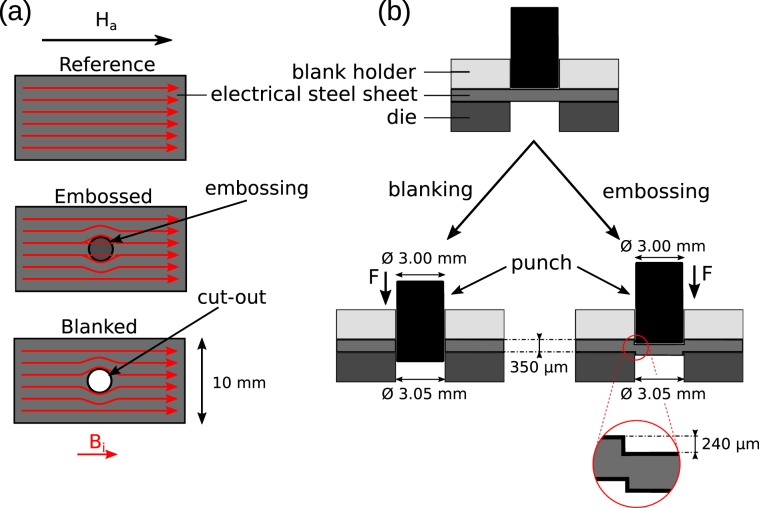
(**a**) Top view of a sketch of electrical steel sheets employing different manufacturing processes. From top to bottom a reference (no embossing or blanking), embossed and blanked electrical steel sheet are shown. The red arrows indicate the flux lines that are induced by the external field *H*_*a*_. (**b**) Side view of a sketch of the manufacturing process of both a blanked and embossed electrical steel sheet. The embossing processes shown is the one used for sample C in Fig. 6.

Taking advantage of the excellent performance of the new nGI setup, we have investigated the pinning of magnetic domains caused by localized stress in non grain-oriented electrical steel sheets containing 2.4 wt% Si. As a result of the pinning of the magnetic domains, the magnetic permeability changes locally. Hence the magnetic flux is deflected from the area influenced by stress, similar to the case of cut-outs introduced in the electrical steel sheets. For a sketch of the expected magnetic flux inside manufactured electrical steel sheets see Fig. 5(a). During the course of the experiment, DFI data from four different electrical steel samples has been acquired. Three samples have been embossed using either a spherical, a conical or a flat punch. The fourth sample was used as a reference and was not embossed. For details see Table 3. A schematic of the embossing process is presented in Fig. 5(b). A summary of the sample properties including the embossing process and the experimental details are given in Sec. 4.5 and Sec. 4.6, respectively.

**Table 3:** Geometry of the samples and parameters of the punches used for embossing the samples.

Sample Name	Sample dimensions	Punch type	Punch depth [μm]
Reference	60 mm × 10 mm × 350 μm	none	none
A	60 mm × 10 mm × 350 μm	sphere with diameter *D*_*s*_ = 2 mm	≈238
B	60 mm × 10 mm × 350 μm	rounded tip with cone angle 110°, *D*_*t*_ = 3 mm	≈306
C	60 mm × 10 mm × 350 μm	cylindrical tip, *D*_*f*_ = 3 mm	≈240

**Figure 6:**
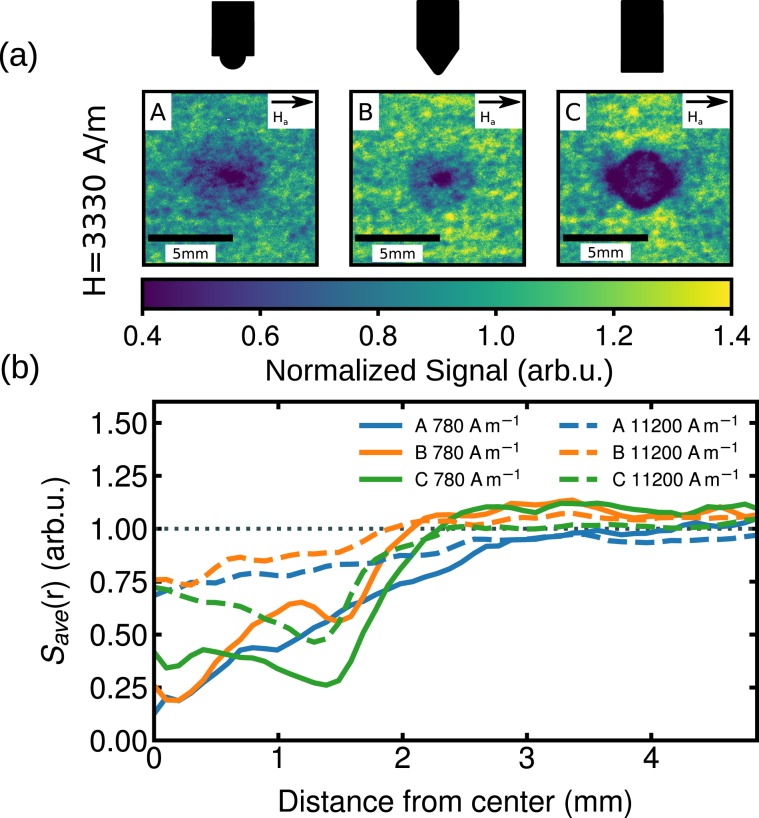
Analysis of the influence of embossing on the scattering contrast. (**a**) DFI-signal of samples A, B and C in an applied magnetic field *H*_*a*_ = 3330Am^−1^. The data is normalized using the reference sample also exposed to *H*_*a*_. A signal smaller than *S* = 1 indicates more scattering in the embossed sample than in the non-embossed reference. (**b**) Normalized signal *S*_*ave*_ (*r*) as obtained by radially averaging the images of samples A (blue), B (orange), and C (green) exposed to two different magnetic fields.

In Fig. 6 the shape of the punches used is shown. The embossing depth has been measured for each sample as explained in Sec. 4.5.

Figure 6(a) shows normalized DFI maps of the embossed samples A, B, and C in a magnetic field *H*_*a*_ = 3330 Am^−1^. To visualize the influence of embossing, the DFI signals of the samples were normalized to the DFI of the reference sample by dividing the respective images pixel by pixel with the reference sample. Hence, a value *S* = 1 denotes no change in the scattering contrast of the embossed sample. Obviously, the three different punches generate different patterns. Figure 6(b) shows plots of radially averaged images of samples A–C, which were exposed to magnetic fields ${H}_{a}\mathrm{=780}\,{{\rm{Am}}}^{-1}$ (solid lines) and *H*_*a*_ = 11200 Am^−1^ (dashed lines). The radially averaged signal is denoted as ${S}_{ave}(r)$. First the results of the measurements at ${H}_{a}\mathrm{=780}\,{{\rm{Am}}}^{-1}$ are presented. Here, sample A exhibits a roughly linear increase of *S*_*ave*_ starting from 0.13 at *r* = 0 to 1 near *r* = 3 mm. For sample B, *S*_*ave*_ attains a value 0.26 at *r* = 0 in *H*_*a*_ = 780 Am^−1^. In contrast to sample A, however, a faster rise of the signal away from *r* = 0 is observed, reaching 1 at *r* = 2 mm. Moreover, the rise of *S*_*ave*_ is not linear, showing a dip at *r* = 1.5 mm. In sample C, *S*_*ave*_ exhibits a minimum near *r* = 1.45 mm. *S*_*ave*_ rises for *r* > 1.45 mm reaching saturation near *r* = 2.2 mm. For *r* < 1.45 mm we observe a slight rise of *S*_*ave*_, indicating that the magnetic field cannot be blocked completely by the barrier formed by the stress, thus allowing the magnetic field to reach the center of the embossed area. For an increased applied magnetic field *H*_*a*_ = 11200 Am^−1^ the signal behaviour changes. Increasing *H*_*a*_ leads to a strong increase of *S*_*ave*_ in sample A, i.e. at the center, *S*_*ave*_ = 0.68. In contrast, the radius where *S*_*ave*_ = 1 is still *r* = 3 mm. In sample B, the dip, seen when applying *H*_*a*_ = 780 Am^−1^, disappears when *H*_*a*_ is increased. Note that *S*_*ave*_ is almost constant for *r* in the range of 0.75 mm to 1.5 mm. The saturation of *S*_*ave*_ is independent of *H*_*a*_ for samples A and B. In sample C increasing *H*_*a*_ causes an increase of *S*_*ave*_ which is, however, not as pronounced as for samples A and B. The differences in sample A and B can be attributed to the different shape of the punch and the embossing process: The deformation of sample A proceeds more gradually because a spherical punch is used. While in sample B the punch causes a secondary residual stress area in the range of 0.75 mm to 1.5 mm due to stronger deformation. The signal of sample C is also not completely circular but shows a deformation along the field direction. It is caused by the interplay between the circular embossing and the unidirectional magnetization direction. Overall, the saturation distance for all samples is largely independent of the applied magnetic field. This is expected, because the distance, where the signal stays constant, depends on the range of the stress and not on the applied magnetic field.

All samples show an increase of the normalized signal *S*_*ave*_ with increasing field indicating that the pinning of the magnetic domain walls due to stress is overcome by *H*_*a*_. The minor deviation of *S*_*ave*_ from unity in the homogeneous area away from the embossing may be caused by small variations in the properties of the base material and differences in the magnetic coupling between the magnetic yoke and the sample.

The deformation introduced by embossing may also influence the grain structure of the electrical steel sheets, which will affect the evaluated nGI-signal. In previous tests it has been shown that the effect of the changed grain structure on the nGI signal is small compared to the effect of the changed magnetic domains due to residual stress^43^. There, an embossed electrical steel sheet has been compared to an embossed and subsequently annealed electrical steel sheet to reduce residual stress. In contrast to the non-annealed sample, the DFI contrast vanishes nearly completely in the annealed sample. The small remaining signal is probably caused by the aforementioned change of the nuclear scattering, or remaining residual stress changing the magnetic domains. It is not possible to completely differentiate between the two effects, as they are strongly connected.

## Conclusion and Outlook

We have presented the specifications and performance of the upgraded neutron grating interferometer at the beamline ANTARES. The adapted geometry of the new nGI allows expanding the range of correlation lengths *ξ* by a factor of 30, i.e. two orders of magnitude in *ξ* can be covered. Moreover, the visibility could be increased by a factor of 3.5 over a large FoV of 71 × 76 mm^2^ reaching almost the theoretical limit. The design of the new setup provides high flexibility in choosing the optimum combination of flux and visibility for each experiment by choosing the appropriate duty cycle for the grating G_0_, i.e. the precision of the data can be optimized with respect to the measurement time. The uncertainty of the DFI signal follows a $\frac{1}{\sqrt{I}}$ dependence for the neutron flux *I* and a $\frac{1}{V}$ dependence for the visibility^44^. As an example, we can use a small duty cycle for experiments, where a high visibility is desired i.e. weakly scattering samples. In case of dynamic measurements a large *DC*_0_ may be chosen to have the necessary detector statistics. Finally, by replacing the phase grating G_1,π_ by G_1,3π_, nGI experiments can be conducted with thermal neutrons (*λ* < 2 Å). Previously, experiments in this wavelength range have not been considered, as the lacking quality of the absorption gratings caused a low visibility, resulting in unreliable measurements.

In a first experiment, we have evaluated the influence of stress triggered by embossing on the local magnetic domain pinning and thus the magnetic permeability of electrical steel sheets using the new nGI. It is shown that embossing with a flat punch yields the most localized stress and hence the most localized decrease of magnetic permeability in the sample. This tailored influence on the magnetic domains allows to guide magnetic fields similarly as the currently used technique of introducing cut-outs. Furthermore, the flat punch provides also the strongest pinning of the magnetic domain walls at higher magnetic fields. Both features indicate that embossing with a flat punch is the best choice to guide magnetic fields in an electrical steel sheet.

In future experiments the effects of the orientation of highest sensitivity of the nGI with regard to (i) the rolling direction of the sample, (ii) the magnetization direction and, (iii) in the case of non radially symmetric punches, the embossing shape will be investigated. The high sensitivity and the wide range of correlation lengths available will allow us to systematically investigate the interplay between the magnetic domains and the resulting scattering signal thus giving us a handle to adjust the embossing process to produce electrical steel sheets with reduced losses. As a result electric motors with improved efficiency may be developed.

Furthermore, the better signal-to-noise ratio of the new nGI allows conducting experiments with shorter exposure times. Hence, experiments analyzing the response of the magnetic domains in an alternating magnetic field, which is used in electric machines, can be performed stroboscopically^45^. Also samples which could not be investigated so far can now be probed. As an example, the vortex lattice domains in superconductors are currently mainly studied in niobium because it exhibits a large scattering contrast between the vortex domains and the Meissner phase^13^. Now, the investigation of the flux line lattice may be extended to other systems such as high *T*_*c*_-superconductors. Despite all of these upgrades the flexibility of the previous nGI-setup with respect to the installation of sample environment and the possibility to perform directional dark-field imaging are maintained.

## Methods

### Design and simulation of new interferometer

During the course of an nGI measurement the variation of the intensity vs. the grating position *x*_*gi*_ is measured for every pixel (*k*,*l*) of the detector^46^:

1 $$I({x}_{{\rm{gi}}},k,l)={a}_{0}(k,l)+{a}_{1}(k,l)cos(\frac{2\pi {x}_{{\rm{gi}}}}{{p}_{{\rm{i}}}}-{\boldsymbol{\phi }}(k,l)),$$

where ${a}_{0}(k,l)$ denotes the mean value of the oscillation, ${a}_{1}(k,l)$ the amplitude, and ${\boldsymbol{\phi }}(k,l)$ the phase. *x*_*gi*_ designates the position of the stepped grating perpendicular to the neutron beam and the lines of the grating^46^. The parameters *a*_0_, *a*_1_ and ***φ*** are determined by fitting the data using Eq. 1. Afterwards, the transmission- (TI), differential phase contrast- (DPCI), and the scattering/dark field image (DFI) are calculated^22^.

The signal-to-noise ratio (SNR) of the DPCI and DFI strongly depends on the visibility $V(k,l)=\frac{{a}_{1}(k,l)}{{a}_{0}(k,l)}$. Therefore, to maximize *V* the following conditions have to be adjusted with respect to each other: (i) the periodicity of the gratings *p*_0_, *p*_1_ and *p*_2_, (ii) their relative distances *L* and *d* in respective parallel alignment (Fig. 1), (iii) the neutron wavelength *λ*, (iv) temporal (bandwidth of the neutron wavelength) and spatial coherence of the neutron beam and (v) the quality of the gratings. (i) - (iii) can be considered to be parameters of the nGI-setup, which are chosen depending on the design of the imaging beamline in which the nGI is installed, as well as the manufacturing limits of the gratings. *L* denotes the distance between G_0_ and G_1_, while *d* denotes the distance between G_1_ and G_2_. The temporal and spatial coherence of the neutron beam (iv) are quantities, which are used to adjust the ratio between flux and visibility, while the absorption of G_0_ and G_2_ and the quality of the etching profile of G_1_ (v) define an upper limit of the achievable visibility. Ideally G_0_ and G_2_ have a binary transmission profile.

The Talbot-Lau interferometer has to adhere to conditions between the inter-grating distances, the grating periods and the wavelength of the neutrons: Firstly, the interference patterns created by each slit of G_0_ have to be projected onto the periodic profile of G_2_ according to the intercept theorem^47^:

2 $$\frac{d}{L}=\frac{{p}_{2}}{{p}_{0}}\mathrm{}.$$

Secondly, the periodicity of G_2_ has to match the periodicity of the interference pattern generated by G_1_, taking into account the magnification effect (*M*) of the conical beam^48^:

3 $${p}_{2}=\frac{L+d}{L}\frac{{p}_{1}}{\eta }=M\frac{{p}_{1}}{\eta }\mathrm{}.$$

Here, $\eta \mathrm{=1}$ and $\eta \mathrm{=2}$ for a *π*/2− and a *π*-shifting phase grating, respectively. $\eta \mathrm{=2}$ is used to account for the frequency doubling effect of the *π*-shifting phase grating. Thirdly, *d* has to be an odd fractional Talbot-distance to allow the observation of an intensity modulation^48^:

4 $$d=n\cdot M\frac{{p}_{1}^{2}}{2\lambda {\eta }^{2}}\mathrm{}.$$

Here, *n* designates the fractional Talbot-order, which is an odd integer $(n\mathrm{=1,3,5,}\ldots )$. A deviation from these three relations leads to the appearance of Moirè fringes or/and to a decrease in visibility and should be avoided. The fractional Talbot-order *n* can be chosen according to the desired sensitivity range and G_1_-G_2_ distance. The new interferometer is designed to allow for the 3^rd^ fractional Talbot-Distance to obtain an increased sensitivity and more space for the sample environment. In Table 1, the effective design parameters of the upgraded nGI are given. Compared to the previous setup, the asymmetry in the grating parameters has been reduced thus relaxing the fabrication requirements for the G_2_ grating.

Although the neutron beam at ANTARES is slightly conical, we performed all calculations for a parallel beam geometry with adapted geometrical parameters, as the results are invariant with respect to different magnification factors^48^. Using grating G_0_, a coherent wave front is created and propagated through the binary phase profile of G_1_ resulting in an intensity modulation according to the Talbot-effect. To exclude edge effects due to the finite size of the propagation matrix the simulation was performed with a kernel of 100 G_1_ periods and only the central intensity modulation was considered for further analysis. The propagation distance G_1_-G_2_ was made equal to the third fractional Talbot-distance induced by a *π*-grating for a wavelength of 4 Å. We treated G_1_ as a pure phase object neglecting the minor absorption by the silicon substrate for simplicity. Then the modulation was convoluted with the binary absorption profiles of G_0_ and G_2_ with the respective duty cycles resulting in an approximately sinusoidal intensity profile. We assumed that G_0_ and G_2_ are binary and fully attenuating gratings, according to previous analysis of their performance in the accessible wavelength range^30^. Since the simulation was sampled very precisely (10 pixel resolution) we extracted the visibility directly from the resulting intensity modulation by taking the difference between the minimum and maximum of the intensity oscillation. To account for the polychromaticity of the neutron spectrum when comparing to our experimental data we calculated and weighted the wavelength-dependent visibility spectrum with a Gaussian-shaped intensity distribution (FWHM as described in Sec. 4.4 below, 0.01 Å bins) as delivered by the neutron velocity selector^49^.

### Correlation length *ξ*

The correlation length $\xi$ is given by^20^

5 $$\xi =\frac{\lambda {L}_{{\rm{s}}{\rm{d}}}^{{\rm{e}}{\rm{f}}{\rm{f}}}}{{p}_{2}},$$

where ${L}_{{\rm{sd}}}^{{\rm{eff}}}$ is the effective sample-to-detector distance. Depending on the placement of the sample with respect to the gratings, ${L}_{{\rm{sd}}}^{{\rm{eff}}}$ is calculated as follows^48^

6 $${L}_{{\rm{s}}{\rm{d}}}^{{\rm{e}}{\rm{f}}{\rm{f}}}=\left\{\begin{array}{cc}(L+d-{L}_{{\rm{s}}{\rm{d}}})\frac{d}{L} & {\rm{f}}{\rm{o}}{\rm{r}}\,{L}_{{\rm{s}}{\rm{d}}} > {\rm{d}}\\ {L}_{{\rm{s}}{\rm{d}}} & {\rm{f}}{\rm{o}}{\rm{r}}{L}_{{\rm{s}}{\rm{d}}} < {\rm{d}}\end{array}\right.$$

from the distance between sample and detector ${L}_{{\rm{sd}}}$. Hence, when placing the sample between G_1_ and G_2_, the correlation length of the nGI can be tuned by changing only the distance between the sample and G_2_ without having to change *λ*. Therefore, the nGI can always be operated at the design wavelength yielding the maximum visibility and SNR.

### Technical realization

All gratings used silicon wafers as substrate material in which the necessary grating lines have been etched by deep reactive ion etching (DRIE). For G_0_ and G_2_ a new fabrication technique involving filling the grooves with Gd powder was used to optimize the quality of the gratings. Both the G_0_-gratings and the G_2_-gratings show a very good binary profile, over the whole area of the grating. Details about the fabrication, the technical data and the transmission performance are reported in^30^.

Four different source gratings with varying duty cycles $(D{C}_{0}s)$ have been produced. As the coherence length of the neutrons rises with decreasing $D{C}_{0}$, a variation allows to optimize flux and visibility as desired for every measurement. The active area of the G_0_ gratings is 65 mm × 65 mm. The positioning system of G_0_ allows to i) rotate the grating around the beam axis (*z*-axis), ii) adjust the effective period by tilting around the *y*-axis, and iii) adjust the total length of the interferometer *S* and the distance between G_0_ and G_1_ by using a translation stage in z-direction in the coordinate system defined in Fig. 1.

Two different phase gratings G_1_, with a circular active area of 110 mm diameter, have been produced. At the design wavelength of 4.0 Å, the ${{\rm{G}}}_{1,{\rm{\pi }}}$ and ${{\rm{G}}}_{1,3{\rm{\pi }}}$ phase gratings cause a phase shift of $\approx \pi$ and $\approx 3\pi$, respectively. In Fig. 2(b) the performance of the two phase gratings as predicted by our simulations is presented. The positioning system also allows for i) a full rotation around the beam-axis, ii) an adjustment of the tilt around the *x*-axis, iii) a translation along the *z*-axis and iv) to perform the stepping scan perpendicular to the grating lines.

The analyzer grating G_2_ has a circular active area with a diameter of 110 mm. Evaluation of the transmission shows that the produced Gd filling has an effective thickness of 16 μm. Due to the small period of the analyzer grating, its fabrication is the most challenging. Previously used methods such as evaporation from the side^29^ or metallic glass filling^31^ have shown to either not provide an ideal binary grating^25,30^ or a limitation of the reproducible area $(\approx 1\,{{\rm{cm}}}^{2})$^31^, respectively. Due to the increased distance *d* even bulky sample environments can now be placed between G_1_ and G_2_. In many cases this new geometry reduces the minimum distance between sample and detector, allowing for a better spatial resolution without the need to decrease the neutron flux by reducing the diameter of the pinhole.

### Experimental procedure for the evaluation of the visibility *V*

We quantified the wavelength dependence of the visibility of the nGI using the four different *DC*_0_. For this evaluation, the ${{\rm{G}}}_{1,\pi }$ phase grating was used. The velocity selector installed at ANTARES has a minimum wavelength of 3 when aligned parallel to the direction of the neutron beam. To access wavelengths between 1.6 Å and 3.0 Å the selector was tilted by 5° which increases the wavelength spread from $\frac{\varDelta \lambda }{\lambda }\mathrm{=10} \%$ to 20%. Additionally, the wavelength dependence of the visibility of the new setup has been acquired for the ${{\rm{G}}}_{1,3{\rm{\pi }}}$ phase grating using a $D{C}_{0}$ of 0.28. The nGI was placed at the optimal distances given for the nGI in Table 1. As a reference, the wavelength dependence of the previous setup described in^22^ has been acquired. The field of view for all scans was 71 mm × 76 mm, which is a limitation of the employed detector. An L/D-ratio of 250 was used and the scintillator thickness of 100 μm resulted in a spatial resolution of approximately 100 μm.

### Specimens

Electrical steel sheets of the identical grade of material have been thoroughly categorized in^50^, from which the values have been reproduced. The chemical composition of the samples is given in Table 4. The sheets were prepared at the Chair of Metal Forming and Casting of the Technical University of Munich as described in^43^. The initial geometry of the samples was chosen to be 90 mm × 15 mm × 350 μm. This allowed to simultaneously emboss the sample and blank locating holes, which were later used to align the samples during the erosion process. The long edge of the sample was parallel to the rolling direction. All embossings have a nominal depth of 200 μm. After embossing, the samples were eroded to a length of 60 mm. This length is imposed by the magnetic yoke^33^ used for the experiment. The width was arbitrarily chosen to be 10 mm. The thickness of 350 μm has been used to prevent saturation of the DFI-signal. For embossing and blanking, a Schuler stamping press CSP 100 with ServoDirect Technology was used. To recover the exact geometry of the embossing, the samples were scanned in 3 dimensions using a Keyence VK-X100 Series Shape Measurement Laser Microscope. The results are reproduced Table 3.

**Table 4:** Chemical composition of the electrical steel sheets.

Element	Fe	C	Si	Mn	P	S	Cr	Al
(wt%)	97.00	0.02	2.42	0.16	0.02	0.01	0.03	0.34

### Experimental parameters for analyzing the stress in electrical steel sheets

The electrical steel sheets were placed in a magnetic yoke allowing to apply a maximum magnetic field ${H}_{a}\mathrm{=11200}\,{{\rm{Am}}}^{-1}$^33^. The rolling direction of the sample, the magnetic field direction of the yoke and the sensitivity direction of the nGI were chosen to be parallel. A correlation length of 1.865 μm was used for the experiment. This correlation length is a result of the distance (62 mm) between sample and G_2_ and the chosen wavelength of 4.0 Å. At this wavelength and using $D{C}_{0}\mathrm{=0.28}$ a visibility of 0.69 has been used.

For each magnetic field strength an nGI-scan was performed by moving G_1_ in ten equidistant steps over one half period, resulting in a movement of the interference pattern by one period at G_2_. At each of the ten steps three images with an exposure time of 10 s were taken, resulting in a total exposure time of 300 s for one scan. The effective pixel size of the detector system was set to 33 μm × 33 μm, resulting in a field of view of 71 mm × 76 mm. A LiF scintillation screen, with a thickness of 100 μm has been used in the detection system. The L/D ratio of the instrument was set to 250. These parameters resulted in a spatial resolution of approximately 100 μm.

## Data Availability

The datasets generated during and/or analysed during the current study are available from the corresponding author on reasonable request.
